# Virtual reality as an assessment tool in neurorehabilitation: a scoping review of current evidence and future directions

**DOI:** 10.1186/s13102-025-01439-1

**Published:** 2025-12-01

**Authors:** Mohammed M. Alrashidi, Ahmed S. Alanazi, Shahad Alkhannani, Amirah Alharbi, Shahad Almuayrifi, Ayman A. Alhammad, Saad A. Alhammad, Abdulrhman S. Mashabi

**Affiliations:** 1 Physical Therapy Department, College of Medical Rehabilitation Sciences, Taibah University, Madinah, Saudi Arabia; 2 Department of Rehabilitation Health Sciences, College of Applied Medical Sciences, King Saud University, Riyadh, Saudi Arabia

**Keywords:** Virtual reality, Assessment, Neurorehabilitation, Psychometrics, Usability, Mixed reality

## Abstract

**Background:**

Virtual reality (VR) technologies are increasingly applied in neurorehabilitation, but most research has focused on their therapeutic applications rather than assessment potential.

**Objective:**

The aim of this scoping review was to map the evidence on VR-based assessment tools in neurorehabilitation, including types of tools, targeted neurological conditions, technological specifications, and research gaps.

**Methods:**

A scoping review was conducted and reported in accordance with PRISMA-ScR. The protocol was prospectively registered in PROSPERO (CRD420251113260). Searches of PubMed, Scopus, Web of Science, The Cochrane Central Register of Controlled Trials (CENTRAL), IEEE Xplore, and grey literature were performed from inception to July 2025. Eligible studies involved VR for assessment purposes in neurological populations and reported validity, reliability, usability, or assessment performance. Data were charted and synthesised thematically.

**Results:**

Sixteen studies met the inclusion criteria. Most adapted established assessments such as the Box and Block Test, Action Research Arm Test, or Virtual Peg Insertion Test into VR formats. Populations included stroke, multiple sclerosis, Parkinson’s disease, cerebral palsy, spinal cord injury, and movement disorders. VR systems ranged from custom-built motion-tracking platforms to commercial head-mounted displays with controllers or optical hand-tracking. Convergent validity ranged from moderate to excellent (*r* = 0.65–0.99), with optical tracking and mixed reality systems (*r* > 0.90) generally outperforming controller-based approaches (r ≈ 0.65). Usability was often rated highly but varied across conditions and modalities, with some studies reporting higher usability ratings for physical tasks compared to VR. Key gaps included limited normative datasets, heterogeneous hardware/software, and minimal longitudinal evaluations.

**Conclusions:**

VR-based assessments in neurorehabilitation remain a developing field. Preliminary evidence suggests good validity, usability, and feasibility, with potential to provide richer performance data that augment conventional assessment. However, large-scale, standardised, and condition-specific studies are required before widespread clinical implementation.

**Supplementary Information:**

The online version contains supplementary material available at 10.1186/s13102-025-01439-1.

## Introduction

Accurate and timely assessment of motor function is essential to optimising rehabilitation outcomes in people with neurological conditions. Assessment tools in neurorehabilitation serve as a foundation, enabling physiotherapists to quantify baseline impairments, guide intervention planning, monitor progress, and make informed decisions about long-term care or return to daily activities [30]. Widely used assessments for upper limb impairments—such as the Box and Block Test (BBT), Action Research Arm Test (ARAT), and Nine-Hole Peg Test (NHPT)—have well-established validity and reliability [19, 31, 36]. However, these assessments rely on manual scoring, provide limited kinematic detail, and are restricted by the ceiling effect [18, 25]. Such shortcomings reduce sensitivity to subtle functional changes over time, compromise ecological validity, and constrain opportunities for frequent or remote monitoring [40].

In recent years, virtual reality (VR) technologies have emerged as promising tools for rehabilitation. By simulating real-world or relevant tasks in a controlled environment, VR can combine the standardisation of traditional assessments with enhanced data capture through built-in sensors [29]. Advances in VR technology—such as head-mounted displays (HMDs), optical tracking, and haptic feedback—enable real-time quantification of movement kinematics, reaction times, force generation, and even cognitive–motor integration [13]. Equally importantly, VR allows assessments to be administered in immersive and engaging contexts, which may improve patient motivation and adherence [21]. Collectively, these features open new avenues for VR not only as a therapeutic intervention but also as an assessment platform with the potential to augment conventional assessments.

Despite these capabilities, most VR research in neurorehabilitation has focused on its therapeutic applications [3]. Far fewer studies have investigated VR as an assessment tool, where its ability to capture detailed, objective, and ecologically valid performance data could be transformative. Therefore, there is a need to systematically map how VR has been applied in neurorehabilitation assessments, the populations and conditions it has targeted, and the gaps that remain unaddressed. Accordingly, the aims of this scoping review were to: (1) identify the types of VR-based assessment tools used in neurorehabilitation; (2) determine the neurological conditions they assess (e.g., cerebral palsy, stroke, Parkinson’s disease); (3) map their technological specifications (e.g., VR system type, hardware and software used); and (4) highlight existing gaps and future research directions in VR-based assessments.

## Materials and methods

This scoping review followed the five stages outlined by Pham et al. [28], Arksey and O'malley [4]: (1) identifying the specific query or problem to be investigated,(2) identifying pertinent research papers; (3) selecting appropriate studies; (4) organising the data; and (5) compiling, summarising, and presenting the findings. In addition, the review was conducted in accordance with the Joanna Briggs Institute (JBI) methodology for scoping reviews [27] and reported in line with the PRISMA-ScR reporting guidelines (refer to Appendix 1) [39]. The protocol of this scoping review was prospectively registered on PROSPERO (CRD420251113260) https://www.crd.york.ac.uk/PROSPERO/view/CRD420251113260. The protocol specified the PCC framework (Population: individuals with neurological conditions of any age; Concept: VR–based tools or systems used for the assessment of motor, cognitive, or functional performance; Context: clinical, laboratory, or rehabilitation research settings). The inclusion and exclusion criteria, along with planned databases, were defined accordingly. The review was conducted in full accordance with the protocol, and no deviations or amendments occurred during its execution.

### Guiding research questions

To develop a comprehensive understanding of the role of VR as an assessment tool in neurorehabilitation, this review was guided by the following questions:

1. What types of VR-based assessment tools have been developed and used in neurorehabilitation?
2. Which neurological conditions are most frequently assessed using VR-based assessment tools?
3. What are the technological specifications (e.g., VR system type, hardware, software) of these assessment tools?
4. What gaps exist in the current literature on VR-based assessments, and what directions should future research take?

### Identification of relevant studies

A comprehensive literature search was conducted across multiple electronic databases: EMBASE (via Ovid), Medline (via Ovid), PubMed, Web of Science, the Cochrane Central Register of Controlled Trials (CENTRAL), IEEE Xplore, and relevant grey literature sources, including ERIC, ICTRP, EU-CTR, ClinicalTrials.gov, and EThOS databases. Both Medline and PubMed were searched to ensure comprehensive coverage, as PubMed often includes newly accepted and ahead-of-print records that are not yet indexed in Medline. The search covered the period from database inception to July 2025. Specific keywords, Medical Subject Headings (MeSH), and Boolean operators (AND/OR) were used to combine terms (Appendix 2).

The search strategy was first developed and tested in PubMed, then adapted for use in the other databases, with modifications as needed. The full PubMed search strategy is reported in Appendix 2. Adapted strategies for Scopus, Web of Science, CENTRAL, IEEE Xplore, and EMBASE/Ovid are provided in Appendix 3. In addition, reference lists of relevant papers were manually screened to identify additional eligible studies not captured by the database searches. No attempts were made to contact authors for supplementary sources.

### Study selection process

Studies were eligible for inclusion if they:

1. Examined the use of VR as an assessment tool in neurorehabilitation.
2. Involved participants with neurological conditions such as cerebral palsy, stroke, traumatic brain injury, multiple sclerosis, spinal cord injury, or Parkinson’s disease.
3. Studies involving healthy participants or healthcare professionals were included when these cohorts were integral to validation, usability testing, or direct comparison with neurological cohorts.
4. Did not restrict study design (e.g., experimental, observational, pilot, feasibility studies, or case reports).
5. Were published in English due to limited availability of translation resources for non-English studies.

Studies were excluded if they:

1. Focused solely on VR-based interventions without an assessment application.
2. Did not report assessment measures or assessment outcomes.
3. Were review articles, opinion pieces, or abstracts without available full texts.

Search results were exported to an Excel spreadsheet for management and screening. Duplicates were identified and removed using a combination of Excel filtering and manual review, based on matching fields (author, title, year, and DOI). After removing duplicates, three independent reviewers (S. Alkhannani, A. Alharbi, and S. Almuayrifi) screened titles and abstracts against the predefined inclusion criteria to identify potentially relevant studies. Full texts of these studies were then retrieved and assessed for eligibility, with inclusion and exclusion criteria reapplied. Reasons for exclusion were documented at the full-text stage. Any disagreements were resolved through discussion or consultation with other reviewers (M. Alrashidi and A.S. Alanazi).

### Data charting process

Three independent reviewers extracted data using a predefined Excel spreadsheet, working in parallel to minimise selection bias. The lead author subsequently cross-checked all extracted data against the full texts to ensure accuracy. This process followed the guidance for conducting scoping reviews outlined by Peters et al. [27].

The data extraction template captured the following key elements from each study:

- ▪ Authors and year of publication
- ▪ Population and sample size
- ▪ Age (mean ± standard deviation)
- ▪ Psychometric properties tested
- ▪ Technology specifications
- ▪ Outcome measures
- ▪ Key findings

Any disagreements regarding extracted data were resolved through discussion and, when necessary, by revisiting the full texts. This process ensured a thorough and reliable extraction, providing a robust foundation for subsequent analysis. No critical appraisal of the included studies was undertaken, as such appraisal is not essential for scoping reviews and is not a mandatory component [4, 39].

### Synthesis of results

Findings from the included studies were synthesised descriptively to map the current use of VR as an assessment tool in neurorehabilitation. The narrative synthesis was guided by the review objectives and involved grouping extracted data into thematic categories:

- ▪ Types of VR-based assessment tools
- ▪ Neurological conditions assessed
- ▪ Technological specifications
- ▪ Identified research gaps

## Results

### Study selection

**Fig. 1:**
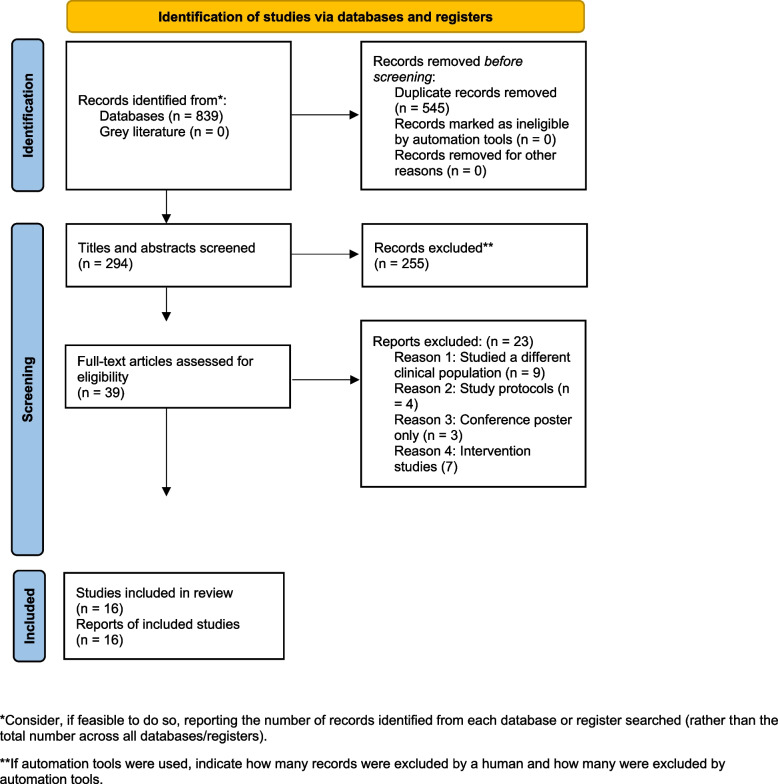
PRISMA flow diagram of the selection process

A total of 839 studies were identified through the systematic search of electronic databases. No eligible records were identified from grey literature sources, which may indicate a limited number of unpublished or non-indexed studies addressing this topic. After removing duplicates, 294 articles were screened by title and abstract, of which 39 were assessed in full text. Twenty-three studies were excluded at the full-text stage for the following reasons: different clinical population (*n* = 9), study protocols (*n* = 4), conference posters only (*n* = 3), and intervention studies (*n* = 7). Sixteen studies met the eligibility criteria and were included in the review. Figure 1 presents the PRISMA flow diagram outlining the study selection process.

### Study characteristics

Table 1 provides a summary of the 16 studies included in this review, which collectively examined 836 participants across a range of populations, including healthy participants, stroke survivors, people with multiple sclerosis, Parkinson’s disease, cerebral palsy (CP), children with movement disorders (e.g., dystonia, chorea, chorea-dystonia), spinal cord injuries, unilateral spatial neglect, and athletes at risk for concussion. Ages ranged from 5 to 74 years, with several studies including both paediatric and adult participants. Sample sizes varied considerably, from as few as 4 participants to as many as 242. The vast majority (14 out of 16) of the included studies were published within the last 10 years.

**Table 1:** Characteristics of the included studies

Authors	Population Total number (*n* = female)	Age (years) (mean ± SD)	Psychometric properties tested	Technology specifications	Outcome measures	Key findings
Burton et al. [6]	Participants with stroke 30 (*n* = 8) Healthy participants 25 (*n* = 15) Healthcare professionals 11 (PTs = 5, OTs = 4 and MDs = 2) (*n* = NR)	Participants with stroke (59.8 ± 10.87) Healthy participants (46.2 ± 23.23) Healthcare professionals (30 ± 7.3)	Validity	Oculus Meta Quest-2 HMD	ARAT and ARAT-VR	The researchers in this study evaluated two versions of the test—ARAT-13 and ARAT-19. Strong correlations were found between the ARAT-VR and both versions: ARAT-13 (*r* = 0.84) and ARAT-19 (*r* = 0.83), indicating comparable performance across the different formats. Additionally, the tool demonstrated excellent usability (SUS = 82.5 [range 75–90]) and outstanding test–retest reliability (ICC = 0.99; *p* < 0.001). Healthcare professionals rated the ARAT-VR as equivalent to the classical version in all tasks except for the marbles subtest, which was perceived as more challenging in the virtual format
Cho et al. [7]	Participants with stroke 9 (*n* = 4)	67 ± 8	Validity	Oculus Rift HMD	Classical BBT and VR version	All participants completed both the BBT and VR-BBT, but block counts were significantly lower in the virtual version for both hemiplegic and non-hemiplegic sides (*p* < 0.001). Strong correlations were observed between BBT and VR-BBT block counts for the non-hemiplegic (*r* = 0.904, *p* = 0.001) and hemiplegic sides (*r* = 0.788, *p* = 0.012), as well as between ratio percentages (*r* = 0.860, *p* = 0.003)
Dong et al. [8]	Participants with stroke 16 (*n* = 5) Healthy participants 113	Participants with stroke 67.88 ± 10.93 Healthy participants (*n* = NR)	Validity, reliability	Oculus Rift HMD	VR version of BBT	The VR-BBT demonstrated strong validity indicators and good test–retest reliability (ICC = 0.75, 95% CI: 0.65–0.83). It provided an engaging method for assessing upper-extremity motor function using kinematic metrics, and the integration of haptic feedback enhanced the precision of upper-limb performance assessment
Everard et al. [10]	participants with stroke 22 (*n* = 5) Healthy participants 23 (*n* = 13)	Participants with stroke (64 ± 10.9) Healthy participants (47 ± 23.9)	Validity	Oculus Quest-1 HMD	Classical BBT and VR version	Results showed strong correlations between the classical BBT and VR-BBT scores in stroke patients for paretic (*r* = 0.89; *p* < 0.001) and non-paretic hands (*r* = 0.76; *p* < 0.001), and in healthy participants for dominant (*r* = 0.58; *p* < 0.01) and non-dominant hands (*r* = 0.68; *p* < 0.001)
Everard et al. [9]	Participants with stroke 21 (*n* = 6) Healthy participants 21 (*n* = 10)	Participants with stroke (65 ± 7.2) Healthy participants (56 ± 11)	Validity, reliability, usability	Oculus Quest-1 HMD, interactive tablet (REAtouch)	Three versions of the BBT: immersive VR with controllers, immersive VR with hand-tracking and with mixed reality	Healthcare professionals rated the BBT version of VR hand-tracking and BBT mixed reality as equally difficult compared to the traditional BBT, while VR-BBT with controllers was perceived as more difficult. In terms of convergent validity, VR-BBT with hand-tracking and classical BBT and its VR version showed strong correlations with the traditional BBT (*r* = 0.94 and *r* = 0.95, respectively), whereas BBT VR with controllers showed a weaker correlation (*r* = 0.65). Usability ratings were excellent for both VR- BBT with hand tracking and mixed reality BBT, with median SUS scores of 83
Gracia-Ibanez et al. [12]	SCI patients 4 (*n* = 0) Healthy individuals 9 (*n* = 8)	SCI patients (34.75 ± 13.07) Healthy individuals (33.33 ± 13.12)	Reliability	LMC and a monitor screen	Classical BBT and VR version	Both groups showed greater trajectory length and velocity peaks in the VR-BBT, with more pronounced changes in SCI patients. SCI participants also had increased finger/thumb flexion and higher muscle activity, indicating impaired coordination and compensatory overactivation, while healthy individuals showed lower EMG activity and more consistent movement patterns. These results underline the likely value of VR-BBT in detecting subtle differences in the dynamic performance of manual dexterity
Kanzler et al. [14]	Participants with MS 31 (*n* = 15) Healthy participants 27 (*n* = 12)	Participants with MS (56 ± 19.5) Healthy participants (30.5 ± 15.5)	Validity, usability	Commercial haptic device, a custom-made handle and a monitor	VPIT, VPIT-2H and PPIT	Intra-participant variability increased with task complexity and was higher in VR, but this did not affect measurement error or reliability. The complex VR task showed greater responsiveness to changes in MS patients, while both VR and physical tasks maintained good clinimetric properties. However, patients rated the physical version as more usable
Lambercy et al. [17]	Participants with MS 10 (*n* = 6) Healthy participants 8 (*n* = 4)	Participants with MS (47.40 ± 3.89) Healthy participants (48.63 ± 4.58)	Validity	Commercial haptic device, a custom-made handle and a monitor	VPIT	Upper limb movements in MS patients were significantly slower, less smooth, and less straight than those of healthy controls. Completion time on the VPIT showed a strong correlation with the conventional NHPT (*r* = 0.658, *p* < 0.01). Additionally, frequency analysis enabled the detection and quantification of intention tremor in the 3–5 Hz range. These preliminary findings demonstrate the feasibility of using the VPIT with individuals with MS and highlight its potential as a tool for evaluating upper limb motor function
Menici et al. [22]	Children with MDs (dystonia, chorea, chorea-dystonia, CP) 16 (*n* = NR)	10.68 ± 3.62	Usability	VRRS based on augmented feedback	VRRS	This pilot study highlights the potential of the VRRS system, in enhancing the rehabilitating postural control and dynamic balance in children with MDs
Oña et al. [23]	Participants with PD 9 (*n* = 1)	71.89 ± 3.66	Validity, reliability	LMC and Oculus Rift HMD	VR version of BBT	Statistical analysis showed that the performance data from the VR-BBT strongly correlated with the well-validated classical BBT and demonstrated high test–retest reliability. These findings support the use of the VR-BBT as a valid and reliable tool for monitoring manual function improvements
Oña et al. [24]	Participants with PD 20 (*n* = 3)	74.35 ± 0.94	Validity	LMC and Oculus Rift HMD	Classical BBT and VR version	The VR-BBT showed strong correlations with classical BBT scores and PD severity (Hoehn and Yahr scale), indicating its potential as a reliable tool for tracking health improvements in PD patients. Authors concluded that the VR-BBT system demonstrated high usability and acceptability, as rated by both clinicians and patients
Perez-Nombela et al. [26]	Children with UCP 7 (*n* = 0)	5.1 ± 1.6	Feasibility, validity	LMC	Classical BBT and VR version	Children scored higher in classical BBT. In the last assessment, children reached 28.17 (SD:6.31) blocks per minute in the classical BBT and 9.00 (SD:5.90) in the VR- BBT
Rosselli et al. [32]	Participants with stroke 37 (*n* = 20) 37 children with CP (*n* = 24) Healthy Participants 205 (*n* = 75)	Participants with stroke (61.7; SD = NR) Children with CP (9.6; SD = NR) Healthy participants (23.31; SD = NR)	Validity	Interactive tablet (REAtouch)	Classical BBT and VR version	The study findings show that the VR-BBT is the most suitable for directly assessing manual dexterity using the REAtouch® device, with potential applications in telerehabilitation. The authors emphasized the need to establish normative data to enable reliable comparisons
Slobounov et al. [34]	55 student-athletes at risk for concussion (*n* = 0)	19.5 (Range 18–25)	Validity	Rear-projected 3D stereo display (6 × 8 ft) operating at 200 Hz, integrated with an AMTI force platform and Flock of Birds motion-analysis system	AMTI force plate and motion tracking (Flock of Birds)	Visual field motion caused long-lasting balance destabilization in concussed individuals but not in controls, even when standard balance tests showed no deficits
Thomasson et al. [37]	USN 39 (*n* = 14)	60.1 ± 7.9	Validity, sensitivity	Oculus Rift HMD	Interactive custom-built VR tasks	Voxel lesion-symptom mapping showed that the deficits identified in VR were linked to damage in the insular and temporal cortex, areas involved in spatial processing. These findings suggest that the immersive VR task is an effective and sensitive tool for detecting mild to severe signs of USN in the extra-personal space
Tobler-Ammann et al. [38]	Participants with stroke 31 (*n* = 8)	62.7 ± 15.1	Validity, reliability	Commercial haptic device, a custom-made handle and a monitor	VPIT, NHPT and BBT	The authors concluded that the VPIT is a promising tool for assessing upper limb function in stroke patients, especially when more detailed evaluation is needed beyond what the traditional NHPT and BBT offer. Although the test can be challenging and shows high variability between trials, a proper orientation helps patients become familiar with it. Once familiarized with the test, the VPIT provides more objective and detailed insights than traditional hand function assessments

*Abbreviations*: *PTs* Physiotherapists’, *OTs* Occupational therapists, *MD* Medical doctor, *SD* Standard deviation, *VR’* Virtual reality, *UCP* Unilateral cerebral palsy, *BBT* Box and block test, *NHPT* Nine-hole peg test, *VPIT* Virtual peg insertion test, *VPIT-2H* A virtual reality version of virtual peg insertion test with reduced task complexity, *PPIT* Physical peg insertion test, *ARAT* Action research arm test, *VRRS* Virtual reality rehabilitation system, *PD* Parkinson’s disease, *MS* Multiple sclerosis, *MDs* Movement disorders, *SCI* Spinal cord injuries, *USN* Unilateral spatial neglect, *r* Pearson’s correlation coefficient, *ICC* Intraclass correlation coefficient, *SUS* System usability scale, *EMG* Electromyography, *HMD* Head-mounted display, *LMC* Leap motion controller, *NR* Not reported

Data are presented as reported in the original studies. Not all studies provided complete statistical indices (e.g., ICCs, CIs, SEM/SDC, AUC) or demographic details (e.g., sex breakdown, SDs). Where quantitative metrics were unavailable, findings are summarized narratively

Most studies investigated virtual adaptations of well-validated physical assessments, for example the BBT [7–10, 12, 23, 24, 26, 32], the ARAT [6], and the VPIT [14, 17, 38]. Other tools included VR balance assessments and concussion assessments [34], custom-built VR environments and tasks for unilateral spatial neglect [37], and the Virtual Reality Rehabilitation System (VRRS) for children with movement disorders [22].

The VR-based tools targeted a variety of conditions, most commonly stroke [6, 7, 9, 10, 38], multiple sclerosis [14, 17], Parkinson’s disease [23, 24], CP [26], spinal cord injury [12], and children with movement disorders [22]. Some studies included healthy participants for validation purposes [10, 32] or healthcare professionals for usability evaluation [6].

Technological specifications varied, with hardware ranging from bespoke setups using motion tracking systems (e.g., Flock of Birds, AMTI force plates) [34] to commercially available HMDs with hand-held controllers [9] or optical hand-tracking [9]. Several studies explored mixed-reality implementations [9] or integrated electromyography to capture muscle activity [12]. Usability ratings were generally high, with System Usability Scale (SUS) scores often exceeding 80 [6, 9, 24].

Few studies addressed long-term clinical utility or integration into routine workflows. Normative data for VR-based assessments were largely absent, and differences in interaction modalities (controller-based vs. hand-tracking) sometimes affected performance outcomes [9].

## Discussion

The aim of this scoping review was to map the evidence on the use of VR as an assessment tool in neurorehabilitation, highlighting a rapidly evolving yet still emerging field. Across the included studies, the predominant approach was to adapt established clinical assessments—such as the BBT [7–10, 12, 23, 24, 26, 32], the ARAT [6], and the VPIT [14, 17, 38]—into VR or mixed-reality formats. These adaptations generally demonstrated convergent validity with traditional measures and were often rated highly for usability; however, usability varied across conditions and modalities, with at least one study (multiple sclerosis cohort) reporting higher ratings for physical tasks compared to VR. Equally importantly, the strength of validity varied by modality, with weaker correlations observed in controller-based tasks compared to optical hand-tracking or mixed-reality systems. It is important to note that the studies included in this review primarily evaluated assessment-related properties (e.g., validity, reliability, feasibility) rather than diagnostic accuracy metrics; therefore, while VR-based assessments may hold future potential for diagnostic applications, the current evidence base should be interpreted within the scope of assessment rather than diagnostic performance.

The included studies highlighted several advantages of VR-based assessments over conventional tools, particularly the ability to capture detailed kinematic data, quantify subtle deficits, and conduct assessments in more engaging environments. This aligns with broader literature suggesting that VR’s potential lies not only in replicating physical tests but also in enhancing assessments with richer, objective metrics that may be sensitive enough to detect early subclinical impairments [1, 41]. Equally importantly, the included studies generally demonstrated convergent validity across tools and conditions**,** although the strength of correlations varied depending on the assessment modality and population. This suggests that VR can serve as a clinically valuable assessment medium with potential diagnostic relevance, rather than merely a novelty add-on. Additionally, many VR platforms—such as Meta Quest and HTC Vive HMDs—captured granular movement data beyond what traditional stopwatch-and-score methods allow, offering potential for improved assessments sensitivity and richer clinical insights [35].

Another advantage is the broad applicability of VR assessments across diverse neurological populations. The included studies spanned both adult and paediatric cohorts, chronic and acute stages, and a range of motor and perceptual deficits. This breadth shows that VR-based assessments can be adapted to multiple rehabilitation contexts. Usability ratings were also generally high, though varied by condition and modality, reflecting strong overall acceptance and feasibility in real-world practice—an essential step toward translating VR assessments from research into clinical application [33].

The findings indicate that VR-based assessments are already capable of supplementing, and in some cases enhancing, traditional neurorehabilitation assessments. This has implications for clinical practice, as VR-based tools may be particularly valuable where kinematic precision, ecological validity, or patient engagement are priorities [5]. For example, optical hand-tracking systems in VR can quantify grasp movement smoothness in ways that may be more sensitive to mild impairments than timed functional tests alone [20]. However, before routine clinical adoption, there must be clear guidance on which VR systems are validated for which populations, along with training for clinicians to interpret VR-specific metrics and VR use [2, 15]. These findings suggest that integration of VR-based assessments into electronic health records and rehabilitation software ecosystems may be an important future direction to support seamless clinical use, but this remains to be established through implementation studies [16].

However, a key limitation of this scoping review lies in the substantial methodological heterogeneity across the included studies. VR systems ranged from bespoke laboratory-based motion-tracking platforms to widely available commercial head-mounted displays. Similarly, interaction modalities varied considerably, with some studies relying on handheld controllers, others employing optical tracking of body segments, and a smaller number using mixed-reality environments. Outcome measures also lacked consistency, spanning kinematic metrics, cognitive–motor integration tasks, and clinical performance scores. This diversity makes direct comparisons between studies problematic and constrains the ability to draw firm conclusions about the overall validity or feasibility of VR-based assessments. For instance, controller-based interfaces may confound results by introducing fine motor demands, while optical tracking affords a more naturalistic assessment of gross motor performance. Commercial headsets offer greater accessibility and potential for scalability, yet their assessment precision remains less explored compared with custom-built systems.

This heterogeneity precludes quantitative pooling or meta-analytic synthesis of the included evidence. However, optical or marker less tracking systems tended to show stronger usability and ecological validity, as they capture natural movement patterns without imposing additional fine-motor control requirements. In contrast, controller-based systems may underestimate gross motor performance due to the added dexterity component. Custom-built laboratory platforms demonstrated high measurement precision and adaptability for research use, whereas commercial headsets offered greater affordability, portability, and scalability for clinical translation. These findings highlight an inherent trade-off between precision and accessibility that should be considered when selecting or designing VR-based assessment tools.

In addition to heterogeneity, the overall quality of the available evidence must also be considered. The majority of included studies were small-scale pilot or feasibility trials, often with limited sample sizes, a lack of control groups, and an absence of longitudinal follow-up or normative datasets. These methodological limitations further reduce the reliability and generalisability of findings and underscore that the evidence base remains preliminary. Together, these factors highlight the urgent need for greater standardisation in hardware, interaction design, outcome reporting, and study design to enable more robust comparisons and to strengthen the evidence base for future clinical application.

Despite promising preliminary evidence, substantial barriers remain before VR-based assessments can be adopted in routine clinical practice. Cost-effectiveness is a key concern, as many VR systems involve specialized hardware and software that may not be financially viable for widespread use without evidence of added value over existing assessment tools. Equally important is clinician training, since effective deployment requires not only technical competence but also confidence in integrating VR tools into everyday workflows. Without structured training programs and clear clinical guidelines, uptake is likely to be slow and uneven [11].

Integration within existing healthcare systems could also pose challenges. Current clinical pathways, record systems, and diagnostic frameworks are not yet designed to incorporate VR-based assessments, creating uncertainty around interoperability, data management, and regulatory approval. Furthermore, the lack of standardisation across platforms and protocols prevents meaningful comparison of results and limits scalability across centres. These challenges suggest that clinical translation will be gradual rather than immediate. Realistically, routine implementation will require stepwise progress through multicentre validation studies, cost-effectiveness evaluations, and the development of normative datasets and training frameworks. Addressing these priorities is essential to ensure that VR assessments can move from experimental prototypes to feasible, sustainable tools in clinical neurorehabilitation.

Based on the evidence mapped in this review, several priorities warrant focused attention to advance VR-based assessments in neurorehabilitation. In the near time**,** the most immediate needs relate to establishing clinical validity. Greater standardisation of protocols and reporting practices would enhance comparability between studies. This could include clearer documentation of task parameters (e.g., target size, such as the virtual cube or peg in a Box and Block–style or peg insertion task, task duration, or movement constraints), calibration procedures (e.g., workspace setup, reach distance), and core outcome metrics (e.g., movement time, smoothness, accuracy). Developing open or consensus-based reporting templates may help support replication and facilitate future meta-analyses. Transparent reporting of hardware and software specifications, calibration steps, and—where possible—raw or processed data availability would further improve reproducibility. While some device-specific efforts to establish normative datasets exist (e.g., REAtouch), few studies in this review—such as Thomasson et al. [37] and Everard et al. [10]—reported broader normative comparisons, and none provided datasets stratified by age, sex, or impairment severity. The lack of standardised, cross-platform reference data limits the interpretability of patient performance and the ability to distinguish developmental from pathological variability. Establishing shared normative frameworks would therefore represent a meaningful step toward improving the clinical reliability of VR-based assessments. Larger, collaborative studies involving diverse neurological populations are also needed to generate age-, sex-, and impairment-stratified reference values and to determine psychometric properties such as minimal detectable change and minimal clinically important difference. Well-designed cross-sectional and prospective studies could contribute valuable insights in this area. In parallel, short-term longitudinal investigations may help clarify whether VR-derived metrics can predict functional outcomes or responsiveness to rehabilitation interventions.

In longer term, continued technological refinement will be essential. Comparative studies across interaction modalities—such as controller-based input, optical tracking, and mixed reality—could help identify approaches best suited to specific clinical contexts. Integrating multimodal sensing (e.g., electromyography, eye tracking, haptic feedback) may also enrich data capture and enhance ecological validity. Finally, extending VR assessments to telerehabilitation settings offers potential to broaden accessibility and continuity of care, particularly in underserved regions.

There are limitations of the present review that must be acknowledged. First, although a comprehensive search strategy was applied across multiple databases and grey literature, it is possible that relevant studies were missed if they were not indexed, used alternative terminology, or were located outside of the searched platforms, which may have introduced selection bias. Equally important, identifying no eligible studies from grey literature sources may reflect the relatively limited dissemination of unpublished or non–peer-reviewed research in this area, which could restrict the comprehensiveness of evidence capture despite extensive searching. Second, as no formal critical appraisal was undertaken (consistent with scoping review methodology), the methodological quality of the included studies could not be assessed, and the risk of bias remains uncertain. Third, the substantial heterogeneity in VR systems, interaction modalities, study designs, and outcome measures precluded quantitative synthesis and meta-analysis. Furthermore, this underscores that our findings should be interpreted as descriptive and exploratory rather than definitive. Additionally, the review did not chart adverse events such as cybersickness, which are pertinent to clinical feasibility and warrant systematic reporting in future studies. Finally, the review was limited to English-language publications, which may have excluded relevant work reported in other languages.

## Conclusion

The aim of this scoping review was to provide the first comprehensive mapping of VR-based assessment tools in neurorehabilitation. While the field is still developing, the existing body of work offers preliminary evidence showing the potential validity, feasibility, and clinical value of VR assessments across a range of neurological conditions. These findings remain constrained by small sample sizes, heterogeneous populations, and limited replication. Future studies focusing on standardisation, normative data, and longitudinal validation are needed to determine whether VR-based assessments can progress beyond experimental prototypes toward broader clinical application.

## Supplementary Information

Additional file 1

Additional file 2

Additional file 3

## Data Availability

The datasets used and/or analysed during the current study are available from the corresponding author on reasonable request.

## Abbreviations

VR: Virtual Reality
BBT: Box And Block Test
ARAT: Action Research Arm Test
NHPT: Nine-Hole Peg Test
PRISMA: Preferred Reporting Items for Systematic Reviews and Meta-Analyses
HMDs: Head-Mounted Displays
VPIT: Virtual Peg Insertion Test
SUS: System Usability Scale
JBI: Joanna Briggs Institute
