# Supplementary material for: Virtual reality as an assessment tool in neurorehabilitation: a scoping review of current evidence and future directions
Source: BMC Sports Sci Med Rehabil. 2025 Dec 1;18:17. doi: 10.1186/s13102-025-01439-1 (PMC12794368; doi:10.1186/s13102-025-01439-1)
Supplement: Supplementary file 2 — Additional file 2 [file 13102_2025_1439_MOESM2_ESM.docx]

**Appendix 2. PubMed search strategy**

(("virtual reality"[Title/Abstract] OR "VR"[Title/Abstract] OR "immersive reality"[Title/Abstract]
OR "mixed reality"[Title/Abstract] OR "extended reality"[Title/Abstract] OR "XR"[Title/Abstract]
OR "augmented reality"[Title/Abstract] OR "AR"[Title/Abstract])
AND
(diagnos*[Title/Abstract] OR assess*[Title/Abstract] OR evaluat*[Title/Abstract]
OR measurement[Title/Abstract] OR "functional assessment"[Title/Abstract]
OR "clinical assessment"[Title/Abstract] OR "clinical evaluation"[Title/Abstract]
OR "functional evaluation"[Title/Abstract] OR "motor assessment"[Title/Abstract]
OR "motor evaluation"[Title/Abstract] OR "motor performance"[Title/Abstract])
AND
(neurorehabilitation[Title/Abstract] OR "neurological rehabilitation"[Title/Abstract]
OR "stroke rehabilitation"[Title/Abstract] OR "Parkinson* rehabilitation"[Title/Abstract]
OR "multiple sclerosis rehabilitation"[Title/Abstract]
OR "cerebral palsy rehabilitation"[Title/Abstract]
OR "spinal cord injury rehabilitation"[Title/Abstract]
OR stroke[Title/Abstract] OR "cerebrovascular accident"[Title/Abstract]
OR CVA[Title/Abstract] OR "Parkinson* disease"[Title/Abstract]
OR "multiple sclerosis"[Title/Abstract] OR "cerebral palsy"[Title/Abstract]
OR "spinal cord injur*"[Title/Abstract] OR "movement disorder*"[Title/Abstract]
OR "traumatic brain injury"[Title/Abstract] OR TBI[Title/Abstract]
OR "unilateral spatial neglect"[Title/Abstract] OR concussion[Title/Abstract]))
AND (humans[Filter])
