# Supplementary material for: Virtual reality as an assessment tool in neurorehabilitation: a scoping review of current evidence and future directions
Source: BMC Sports Sci Med Rehabil. 2025 Dec 1;18:17. doi: 10.1186/s13102-025-01439-1 (PMC12794368; doi:10.1186/s13102-025-01439-1)
Supplement: Supplementary file 3 — Additional file 3 [file 13102_2025_1439_MOESM3_ESM.docx]

**Appendix 3: Search strategies in databases**

1. **Web of Science**

("virtual reality" OR VR OR "immersive reality" OR "mixed reality" OR "extended reality"

OR XR OR "augmented reality" OR AR)

AND TS=(diagnos* OR assess* OR evaluat* OR measurement

OR "functional assessment" OR "clinical assessment"

OR "clinical evaluation" OR "functional evaluation"

OR "motor assessment" OR "motor evaluation" OR "motor performance")

AND TS=(neurorehabilitation OR "neurological rehabilitation" OR "stroke rehabilitation"

OR "Parkinson* rehabilitation" OR "multiple sclerosis rehabilitation"

OR "cerebral palsy rehabilitation" OR "spinal cord injury rehabilitation"

OR stroke OR "cerebrovascular accident" OR CVA

OR "Parkinson* disease" OR "multiple sclerosis"

OR "cerebral palsy" OR "spinal cord injur*"

OR "movement disorder*" OR "traumatic brain injury"

OR TBI OR "unilateral spatial neglect" OR concussion)

Refined by: DOCUMENT TYPES=(Article) AND LANGUAGE=(English)

1. **Scopus**

TITLE-ABS-KEY("virtual reality" OR VR OR "immersive reality" OR "mixed reality" OR "extended reality"

OR XR OR "augmented reality" OR AR)

AND TITLE-ABS-KEY(diagnos* OR assess* OR evaluat* OR measurement

OR "functional assessment" OR "clinical assessment"

OR "clinical evaluation" OR "functional evaluation"

OR "motor assessment" OR "motor evaluation" OR "motor performance")

AND TITLE-ABS-KEY(neurorehabilitation OR "neurological rehabilitation" OR "stroke rehabilitation"

OR "Parkinson* rehabilitation" OR "multiple sclerosis rehabilitation"

OR "cerebral palsy rehabilitation" OR "spinal cord injury rehabilitation"

OR stroke OR "cerebrovascular accident" OR CVA

OR "Parkinson* disease" OR "multiple sclerosis"

OR "cerebral palsy" OR "spinal cord injur*"

OR "movement disorder*" OR "traumatic brain injury"

OR TBI OR "unilateral spatial neglect" OR concussion)

AND (LIMIT-TO(DOCTYPE, "ar") OR LIMIT-TO(DOCTYPE, "cp"))

AND (LIMIT-TO(LANGUAGE, "English"))

1. **CENTRAL (Cochrane Library)**

("virtual reality" OR VR OR "immersive reality" OR "mixed reality" OR "extended reality"

OR XR OR "augmented reality" OR AR):ti,ab,kw

AND (diagnos* OR assess* OR evaluat* OR measurement

OR "functional assessment" OR "clinical assessment"

OR "clinical evaluation" OR "functional evaluation"

OR "motor assessment" OR "motor evaluation" OR "motor performance"):ti,ab,kw

AND (neurorehabilitation OR "neurological rehabilitation" OR "stroke rehabilitation"

OR "Parkinson* rehabilitation" OR "multiple sclerosis rehabilitation"

OR "cerebral palsy rehabilitation" OR "spinal cord injury rehabilitation"

OR stroke OR "cerebrovascular accident" OR CVA

OR "Parkinson* disease" OR "multiple sclerosis"

OR "cerebral palsy" OR "spinal cord injur*"

OR "movement disorder*" OR "traumatic brain injury"

OR TBI OR "unilateral spatial neglect" OR concussion):ti,ab,kw

Limits: Humans, Trials, English language

1. **IEEE Xplore**

("virtual reality" OR VR OR "immersive reality" OR "mixed reality" OR "extended reality"

OR XR OR "augmented reality" OR AR)

AND (diagnos* OR assess* OR evaluat* OR measurement

OR "functional assessment" OR "clinical assessment"

OR "clinical evaluation" OR "functional evaluation"

OR "motor assessment" OR "motor evaluation" OR "motor performance")

AND (neurorehabilitation OR "neurological rehabilitation" OR "stroke rehabilitation"

OR "Parkinson* rehabilitation" OR "multiple sclerosis rehabilitation"

OR "cerebral palsy rehabilitation" OR "spinal cord injury rehabilitation"

OR stroke OR "cerebrovascular accident" OR CVA

OR "Parkinson* disease" OR "multiple sclerosis"

OR "cerebral palsy" OR "spinal cord injur*"

OR "movement disorder*" OR "traumatic brain injury"

OR TBI OR "unilateral spatial neglect" OR concussion)

Filters applied: Humans, English language, Journals

1. **EMBASE (via Ovid)**

('virtual reality'/exp OR 'virtual reality':ti,ab OR VR:ti,ab OR 'immersive reality':ti,ab

OR 'mixed reality':ti,ab OR 'extended reality':ti,ab OR XR:ti,ab

OR 'augmented reality':ti,ab OR AR:ti,ab)

AND (diagnos*:ti,ab OR assess*:ti,ab OR evaluat*:ti,ab OR measurement:ti,ab

OR 'functional assessment':ti,ab OR 'clinical assessment':ti,ab

OR 'clinical evaluation':ti,ab OR 'functional evaluation':ti,ab

OR 'motor assessment':ti,ab OR 'motor evaluation':ti,ab OR 'motor performance':ti,ab)

AND (neurorehabilitation:ti,ab OR 'neurological rehabilitation':ti,ab

OR 'stroke rehabilitation':ti,ab OR 'Parkinson* rehabilitation':ti,ab

OR 'multiple sclerosis rehabilitation':ti,ab OR 'cerebral palsy rehabilitation':ti,ab

OR 'spinal cord injury rehabilitation':ti,ab OR stroke:ti,ab

OR 'cerebrovascular accident':ti,ab OR CVA:ti,ab OR 'Parkinson* disease':ti,ab

OR 'multiple sclerosis':ti,ab OR 'cerebral palsy':ti,ab OR 'spinal cord injur*':ti,ab

OR 'movement disorder*':ti,ab OR 'traumatic brain injury':ti,ab

OR TBI:ti,ab OR 'unilateral spatial neglect':ti,ab OR concussion:ti,ab)

Limits: Humans, English language, Articles
